# A Triadic Neurocognitive Approach to Addiction for Clinical Interventions

**DOI:** 10.3389/fpsyt.2013.00179

**Published:** 2013-12-27

**Authors:** Xavier Noël, Damien Brevers, Antoine Bechara

**Affiliations:** ^1^Psychological Medicine Laboratory, Faculty of Medicine, Université Libre de Bruxelles, Brussels, Belgium; ^2^Department of Psychology, Brain and Creativity Institute, University of Southern California, Los Angeles, CA, USA

**Keywords:** addiction, self-regulation, impulsive system, interoception, decision making

## Abstract

According to the triadic neurocognitive model of addiction to drugs (e.g., cocaine) and non-drugs (e.g., gambling), weakened “willpower” associated with these behaviors is the product of an abnormal functioning in one or more of three key neural and cognitive systems: (1) an amygdala-striatum dependent system mediating automatic, habitual, and salient behaviors; (2) a prefrontal cortex dependent system important for self-regulation and forecasting the future consequences of a behavior; and (3) an insula dependent system for the reception of interoceptive signals and their translation into feeling states (such as urge and craving), which in turn plays a strong influential role in decision-making and impulse control processes related to uncertainty, risk, and reward. The described three-systems account for poor decision-making (i.e., prioritizing short-term consequences of a decisional option) and stimulus-driven actions, thus leading to a more elevated risk for relapse. Finally, this article elaborates on the need for “personalized” clinical model-based interventions targeting interactions between implicit processes, interoceptive signaling, and supervisory function aimed at helping individuals become less governed by immediate situations and automatic pre-potent responses, and more influenced by systems involved in the pursuit of future valued goals.

## Introduction

Once an individual has lost control over drug or non-drug (e.g., gambling) use, rising negative consequences do not necessarily lead to any adjustment in their maladaptive choices and actions (1). Reasons for the transition from controlled use to addiction are numerous and include individual vulnerabilities in attention, reward, emotion, decision-making, self-regulation, pain, and stress [for a review, see Ref. (2)]. Additional reasons include the neurotoxic and neuroadaptive effects of drugs and withdrawal on the central nervous system [e.g., Ref. (3)].

Historically, the research on addiction has attracted studies that focus on only one neural system at a time. For instance, during the 1980s, the research focused on the role of the striatum and mesolimbic dopamine system, as well as the amygdala, in drug reward [e.g., see Ref. (4) for a review]. During the 1990s, the prefrontal cortex attracted most of the research on addiction, and its role in various mechanisms of decision-making and impulse control. More recently, there has been a rising interest in the role of the insular cortex in addiction [e.g., Ref. (4, 5)]. In an attempt to unify the concept of addiction that integrates both experimental and clinical perspectives, we present here the triadic neurocognitive model of addiction, which takes into account all the neural systems that have been implicated historically in the neurobiology of addiction, including “willpower,” the ability to self-control, and the capacity to choose according to long-term, rather than short-term, outcomes [see also (6)]. We argue that in addiction to drugs (e.g., cocaine) and non-drugs (e.g., gambling), the weakened “willpower” associated with most addictive behaviors is the product of an abnormal functioning in the interaction between three key neural and cognitive systems: (a) an amygdala-striatum dependent neural system, which promotes automatic cognitions and habitual actions; we have referred to this neural system as the “impulsive” system, and it becomes hyperactive in states of addiction; and (b) a prefrontal cortex dependent neural system, which is important for decision-making, forecasting the future consequences of a behavior, and inhibitory control and self-awareness; we have referred to this neural system as the “reflective” system, and it may become hypoactive in states of addiction; and (c) the insular cortex, which plays a key role in modulating the dynamics between the “impulsive” and “reflective” systems. More specifically, the (anterior) insula is important for translating bottom-up, interoceptive signals into subjective experiences (e.g., craving), which in turn potentiates the activity of the impulsive system, and/or weakens or hijacks the goal-driven cognitive resources needed for the normal operation of the reflective system. At the cognitive level, the characteristics of the impulsive and reflective neural systems mirror those from the dual-processing accounts; one is fast, automatic, and unconscious and the other is slow, deliberative, and conscious (7–9). Automatic or poorly controllable processes include what has been addressed in the literature as biased attention processing toward addiction-related cues (e.g., a bottle of beer, a slot machine, a syringe), implicit memory associations (e.g., alcohol-pleasant associations), and approach/avoidance responses to addiction-related cues (10). However, habits and impulsive behaviors can be brought under control by controlled processes related to goal-directed actions, at least to some extent. These automatic processes can be modified through the engagement of self-regulation processes. In case of addictive behaviors, a number of executive functions either cool (e.g., working memory) or hot (e.g., redirecting attention from addiction- to non-addiction-related cues, inhibiting addiction-related pre-potent responses) are viewed as compromised and unable to exert an effective control of behaviors elicited by automatic processes [e.g., Ref. (11)].

While this dual-process model may explain many aspects of complex decision-making, self-control, and “willpower” to resist the temptation of addictive stimuli, we argue that the model is not sufficient to explain all aspects of addictive behaviors, especially those related to deprivation from the reward stimulus or withdrawal, stress, anxiety, and other homeostatic disturbances. For example, while an individual with alcoholism may manage to exert self-control over his or impulse to have a drink for a period of time, the mere sight of a bottle, or experiencing a stressful situation, can throw this control completely out of balance, and the old automatic and habit systems take over. We argue that this drastic weakening of control systems brought about by changes in the internal body state of the organism play a key role in modulating the strengths of impulsive and reflective systems, and incorporating this influential role of internal states is not part of the dual-process models. For this reason, we propose a key role for a third neural system, namely the insula [e.g., see its role in nicotine dependence, (12)]. The insula is viewed as a “gate” system involved in the way a person feels at a particular point in time, which responds to homeostatic perturbations (13), and in turn modulates the interaction between these two systems, i.e., the impulsive and reflective systems (4, 5, 14). Specifically, engagement of the insula exacerbates activity of the impulsive system and hijack activity of the reflective system; thus creating a severe imbalance between the two. At the neuroanatomic level, strong afferent projections connect the insula to the ventral striatum (15). In addition, insular hyperactivity during addiction-related cue exposure may result in compromising self-control efficiency. In light of well-documented links between stressor exposure and addiction-related experiences (e.g., relapse), stressor exposure modulate cue reactivity to drug cues [for a review, see Ref. (16)] and thus may dramatically impact the balance between the impulsive and reflective systems. Besides, the imbalance found in individuals struggling with their compulsive behaviors often accompanies blunted sensitivity to non-drug rewards (17); thus making addiction-related activities highly attractive.

Finally, in this article, we will elaborate on clinical model-based interventions targeting implicit processes, interoceptive processing, and supervisory functioning aimed at helping individuals become less governed by immediate situations and automatic pre-potent responses, and more influenced by systems involved in the pursuit of future valued goals. We argue that this pathway may restore some kind of “free will,” thus making the initiation and the maintenance of a change process of addictive behaviors more likely and more effective.

## The Impulsive System, and its Implicit Cognitive Determinants of Addictive Behaviors

Over the course of the development of an addiction, related behaviors become progressively controlled by addiction-associated information that have acquired, through Pavlovian and instrumental learning mechanisms, the property to automatically elicit substance-related (or gambling) actions (18, 19). In this section, we describe some neural and cognitive properties of what we have called “impulsive system,” and focus on some of its properties that are associated with addictive behaviors.

These fast and poorly deliberated responses triggered by competent cues present in the environment (e.g., the view of a bottle of beer by a drinker) depend on basal ganglia neural circuits (20). Critically, the amygdala-striatal (dopamine dependent) neural system is a key structure for the incentive motivational effects of a variety of non-natural rewards (e.g., psychoactive drugs) and natural rewards (e.g., food) (21). This stimulus bound rigid and automatic-habit decision-making system requires little mental elaboration (22), and it is modified by drugs, alcohol, and gambling through changes in the phasic characteristics of dopamine activity in reward signaling, and the tonic function of dopamine levels in permitting and facilitating a large variety of motor and cognitive functions (23, 24). Increased mesolimbic dopamine activity, stimulated by drugs of abuse, reinforces the repetition of behaviors, influencing learning and attentional processes, and the strengthening of associations of reinforcing effects (10, 25, 26). Through intensive practice and operant conditioning processes, instrumental performance (e.g., a rat pressing a lever to receive cocaine) could easily switch from goal-directed action-outcome associations, which requires a representation of the outcome as a goal, to actions more independent of the current value of the goal (27); thus characterizing a state of compulsivity (28). The transition between goal-directed and compulsive behaviors was associated with specific aspects of synaptic structural plasticity in both dorsal (29–31) and ventral striatal regions (31). This transition process is accelerated by the sensitization of dopaminergic systems (32). At the cognitive processing level, the impulsive system processes information automatically/implicitly, which describes a number of properties such as goal independence, absence of intentionality, uncontrollability, lack of awareness of one or more aspects of the process (e.g., stimuli, origins, attributes, behavioral effects), efficiency (effectiveness under processing load), and operation even under time constraints (33).

A vast literature has investigated the relationship between automatic/implicit processes and behaviors and reached the conclusion that those processes could modify action in the absence of any decision (34). For instance, in experiments investigating priming effects, participants who were primed with words such as “wrinkles” or “gray” walked more slowly when leaving the experiment (35). In this case, one could expect that conscious deliberation be not impacted by this experimental condition; thus highlighting a direct implicit cognition/action pathway. In other cases, viewing a beer advertisement in the television may result in making the decision to go to the fridge and grasping a beer, it seems reasonable to posit that some automatic processes resulted in biasing the decision possibly through the signaling of the immediate rewarding aspects of the experience (e.g., pleasant taste of the beer), sometimes at the detriment of differed consequences (e.g., fatigue). In line with this idea, is the experiment showing that altering the effort required to reach foods by manipulating their proximity can increase the selection of easier-to-reach food options (36). With respect to drug use and gambling, addiction-related cues are progressively flagged as salient and grab the addicts’ attention (i.e., attentional bias) (37). The attentional processing of such stimuli trigger implicit “wanting” and generate automatic approach behaviors (10).

Addicts exhibit a modified attentional processing for addiction-relevant stimuli (25). These attentional biases occur at both early and later levels of attentional processing, that is, at both automatic, and at more conscious and deliberate, levels (37). Indeed, early level of attentional processing (e.g., attentional encoding; initial orientation of attention) depends primarily on automatic-habit processes (38, 39), whereas later attentional processes (i.e., maintenance of attention; disengagement of attention) involve higher level of conscious control processes (39). Attentional bias at the level of attentional encoding has been studied with the attentional blink (AB) paradigm (40). The AB phenomenon refers to the observation that the second of two-masked targets (T1 and T2), which appears in a rapid serial visual presentation (RSVP) stream of distracters, is usually poorly identified when it is presented within a short time interval after T1 [e.g., within a several 100 ms; (40)]. Using this task, recent studies [e.g., Ref. (41, 42)] have shown that, in addicts, addiction-related words were less affected by interference of other RSVP items within a short time interval after T1, as compared with neutral words. These results suggest that individuals suffering from addiction are more likely to identify addiction-related words than neutral words under conditions of limited attentional resources, which is consistent with an enhanced attentional bias for addiction cues at the encoding level. Attentional bias at the engagement and the maintenance/disengagement levels of attention has been highlighted through the monitoring of eye-movements during performance of attentional tasks [for a review, see Ref. (37)]. For instance, using a change detection task, Brevers et al. (43) showed that, compared with their controls, problem gamblers were faster to detect gambling-related than neutral-related stimulus change. In addition, these investigators observed that problem gamblers directed their first eye-movements (i.e., attentional engagement) more frequently toward gambling-related than toward neutral stimuli, exhibited more gaze fixation counts (i.e., attentional maintenance) on gambling stimuli and spent more time looking (i.e., attentional maintenance) at gambling-related than neutral stimuli.

Implicit addiction-related association refers to spontaneous associations between addiction-related cues and affective, arousal, motivational representation in memory. This association tends to reveal automatic, impulsive cognitive-motivational mental processes, which are sparsely independent of, or not available to, conscious awareness (10). In other words, implicit association could be defined as an introspectively unidentified (or inaccurately identified) trace of past experience that mediate feeling, thought, or action (44). The Implicit Association Task [IAT; Ref. (45)] is a paradigm commonly used to assess implicit association. In a typical IAT, stimuli belonging to one of four possible categories are presented one by one on a computer screen. On each trial, participants categorize as fast as they can the presented stimulus by pressing one of two keys. The assumption underlying the IAT effect is that two concepts that are more closely related in memory should facilitate responses (faster responses and less errors) when they share the same response key, and impair responses when they do not share the same response key. Hence, they should be faster in the first categorization than in the second. For instance, when classifying names of alcohol or soft drinks (i.e., target stimuli), and positive or negative words (i.e., attribute stimuli), people who hold stronger alcohol-positive affect associations than soft drink positive affect associations should be faster when alcohol and positive words are assigned to one key, and soft drinks and negative words to the second key, when compared to the condition in which soft drinks and positive words are assigned to one key, and alcohol and negative words to the other. Using this task, researches have shown that appetitive associations (i.e., positive, arousal) predict alcohol and substance use (46). For instance, Thush and Wiers (47) observed that positive affect and arousal associations predict the level of drinking in adolescents for the following year.

In addition to attentional bias and implicit association, prominent addiction models posit that automatically activated approach/avoidance tendencies play a critical role in addition. Indeed, the repetition of addiction-related behaviors elicits automatic approach tendencies toward addiction-related cues (10). Several paradigms have been developed to assess this action tendency. Consider, for example, the stimulus-response compatibility task (48), in which participants are instructed, in one block, to move a manikin (i.e., a little man) as fast as they can toward substance-related pictures, and away from neutral pictures (approach substance block). In another block, the task is to move the manikin away from the substance-related pictures and toward neutral pictures (avoid substance block). Through this task, substance abusers (alcohol, cigarettes, marijuana) exhibited relatively fast approach movements toward substance cues (48–50). Hence, by highlighting that addiction-related cues automatically trigger a corresponding impulse, which consists of a behavioral schema to approach it (10), these studies provide strong evidence for the presence of automatic incentive habits in gambling addiction. However, it is important to realize that these tendencies are likely to be highly malleable and context-dependent. For instance, unlike controls, abstaining alcohol-dependent patients revealed a relative avoidance bias rather than relative approach bias and moreover, relapse rates were found to increase as the relative tendency to avoid alcohol increased (51).

Altogether, these cognitive aspects are consistent with the incentive-sensitization theory (19, 52), which suggests that, through repetition of rewarding appetitive experiences, the degree to which addiction-related objects are “wanted,” desired, and their effect anticipated, increases disproportionately when compared with the degree to which they are “liked” (i.e., the actual mood change), and this dissociation may progressively increase with the development of addiction. In addition to the increased salience attributed to cues that predict drug reward, addiction is characterized by relatively reduced sensitivity to natural rewards (17, 53), as seen for instance in cocaine abusers for whom non-addiction rewards generate below normal mesocorticolimbic neural activations as compared to monetary reward (54). Also, while occasional smokers show greater neural responses to money than cigarettes, dependent smokers react equally across conditions (55). We should note here that money reward may be exchanged for smoking or drugs or any other addictive stimulus. For this reason monetary reward seems to exert strong neural responses. Most importantly, addictive stimuli acquire a strong incentive motivational salience (e.g., Robinson and Berridge), which renders it highly more preferable to any other type of reward (except money). This increased reward salience is also evident in animal paradigms. While novelty serves as a strong reward stimulus in animals, those animals that become exposed to drugs begin to prefer the choice of the drug instead of the novelty (56), and even instead of maternal behavior (57), or food (58).

## The Reflective System and Its Supervisory Functions in Addiction

While the habit (or impulsive) system, which is key to generating at least the “wanting” component to seek reward, may explain one important aspect of the behaviors associated with approach behaviors, it is clear that it does not explain how one does control his or her behavior. This function refers to the action of what we called the “reflective system,” which is necessary to control these more basic impulses, particularly when the behavior may not be optimal or advantageous, or is perceived as the incorrect thing to do; and it disallows a more flexible pursuit of long-term goals (17). In this section, we review some evidence showing that addicted individuals have abnormalities in this system and have difficulties in exercising willpower to resist the habits and temptations associated with their compulsive actions.

Self-control can be estimated through the capacity to inhibit pre-potent motor responses. This process refers to the ability to deliberately suppress dominant, automatic responses that are no longer relevant or required. This type of inhibitory control can be indexed by the stop-signal [e.g., Ref. (59)] and go/no-go tasks [e.g., Ref. (60)], which require the subject to withhold simple motor responses when a stop-signal occurs (stop-signal task), or when a no-go stimulus is presented (go/no-go task). Impaired response inhibition performance has been previously highlighted in addicts by using the stop-signal task (i.e., prolonged latency of motor response inhibition), and the go/no-go paradigm (i.e., more errors of commission: subject had to withhold a response but pressed a button instead) [for a review of response inhibition impairment in gambling, opiate, and alcohol addiction, see respectively (17, 61, 62)]. Moreover, results from several brain imaging studies showed that, in drug addicts (63, 64), impaired performance on response inhibition tasks is associated with a hypoactivation in the anterior cingulate cortex, implicated in mechanisms of error detection, and conflict monitoring. Several studies also reported intact response inhibition in addicts [e.g., Ref. (65, 66)]. Nevertheless, this absence of behavioral difference is not necessarily indicative of intact response inhibition processes. For instance, van Holst et al. (67) observed increased dorsolateral and anterior cingulate cortex activity in pathological gamblers who exhibited a similar behavioral performance as their controls on motor response inhibition. In other words, a more effortful strategy (i.e., higher brain activation) was undertaken in pathological gamblers to perform at a similar level as their controls (67). Importantly, disruption in response inhibition processes could lead to abnormal salience attribution directed at addiction-related cues in addicts. In other words, a dysfunction of the inhibitory control system could further exacerbate automatic impulsive processes. For instance, implicit memory associations are a strong predictor of alcohol use in young adults with poor ability of motor response inhibition (68). Moreover, several studies showed that response inhibition deficits affecting drug and gambling addicted persons (69) are thought to accelerate the course of addiction by compromising abstinence from cocaine (70), gambling (71), nicotine (72), alcohol (73), aggravating problem gambling (74), and by increasing attrition from treatment (75).

The action of the “reflective” system is also crucial for choosing according to long-term, rather than short-term, outcomes (6). This function is mediated by paralimbic, medial orbitofrontal, and ventromedial prefrontal structures involved in triggering somatic states from memories, knowledge, and cognition, which allow activation of numerous affective/emotional (somatic) responses that conflict with each other; the end result is that an overall positive or negative signal emerges (76). The impact of somatic responses in addiction has been initially demonstrated in clinical research with patient populations with damage in frontal lobe regions as well as imaging studies that delineate the likely neural basis of each of these functions (76–78). After damage to the ventromedial region of the prefrontal cortex, previously well-adapted individuals become unable to observe social conventions and decide advantageously on personal matters (79). The nature of these deficits revealed that the vmPFC region serves as a link between (a) a certain category of event based on memory records in high order association cortices to (b) effector structures that produce an emotional response (77). Damage to the systems that impact emotion and/or memory compromise the ability to make advantageous decisions (79). The Iowa Gambling Task [IGT; (80)], which was initially developed to investigate the decision-making defects of neurological patients in real-life has been shown to tap into aspects of decision-making that are influenced by affect and emotion (77). This task has been regarded as the most widely used and ecologically valid measure of decision making in this clinical population. One of the reasons for this ecological validity is that performing advantageously on this task requires, as in real-life, dealing with uncertainty in a context of punishment and reward, with some choices being advantageous in the short-term (high reward), but disadvantageous in the long run (higher punishment); other choices are less attractive in the short-term (low reward), but advantageous in the long run (lower punishment). Hence, the key feature of this task is that participants have to forgo short-term benefits for long-term benefits, a process that is presumably severely hampered in drug and gambling addicts (1). Accordingly, performance on the IGT has been shown to be a sensitive measure of impaired decision-making in a diversity of neurological and psychiatric conditions (6). For instance, patients with frontal lesions [e.g., Ref. (80–82)], individuals suffering from substance [e.g., Ref. (83–87)] or non-substance addiction [e.g., Ref. (88, 89)] have demonstrated a preference for short-term gains despite larger net losses while performing the IGT.

In sum, disrupted function of the “reflective” prefrontal cortex could lead to impaired response inhibition and abnormal salience attribution toward high-short-term rewards in addictive individuals. This provides one explanation of addicts’ inability to resist substance (or addiction-related behavior) seeking and taking at the expense of non-addiction-related activities.

## Neural Systems That Intensify Motivation and Weaken Control of Behavior: The Insula

**Figure 1:**
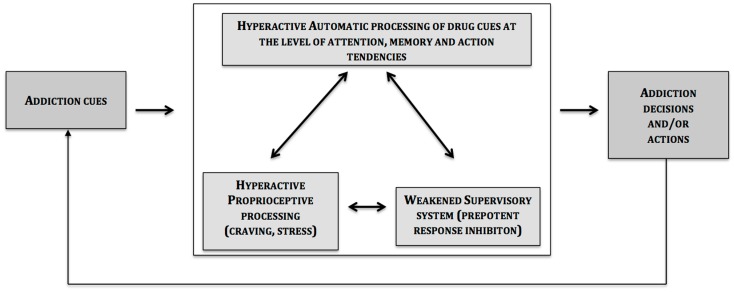
**Schematic representation of the triadic model of addictive behaviors**.

The central message of this article is that the two systems described so far are influenced by interoceptive processes (e.g., drug cravings) (see Figure 1).

At the heart of this phenomenon is the insula in which activation in craving paradigms is a reliable observation (5, 90). However, before discussing further about the specific role of the insula, one should keep in mind that the insula does not work in isolation in the brain, and that in the case of a craving for a certain drug, other regions are activated in cue-reactivity and drug-craving paradigms [for a review, see, Ref. (90)]. These data are in line with the proposal of the present article, which suggests that the insula is connected to brain-related impulsive (amygdalar striatal complex) and reflective (prefrontal cortex) systems; and it modulates their impact on decision and action (4).

The insular cortex has recently emerged as a key neural structure that plays a key role in the formation of interoceptive representation, which is crucial for subjective emotional feelings (5, 13, 91). Interoception refers to the practice of receiving, processing and integrating body-relevant signals with external stimuli of affect on-going motivated behavior (13). According to the notion of embodiment, high-level cognitive and affective processing is grounded in the organism’s sensory and motor experiences (92). Moreover, it has recently been argued that the insular cortex may contribute to the onset and maintenance of addiction by translating interoceptive signals into what one subjectively experiences as a feeling of desire, anticipation, or urge leading to approach behaviors (5, 14, 93). Indeed, highly embodied experience may overwhelm the cognitive control system by providing a highly emotional experience. But also, low levels of embodied experience may not engage the cognitive control system to adjust on-going behavior, which may result, for instance, in high risk-taking (14).

Imaging studies show activity within the insula correlating with the subjects’ rating of urge for cigarettes, cocaine, alcohol, and heroin (5, 13, 93). In addition, insula activation was closely associated with higher levels of nicotine dependence (94) and both attentional bias and the risk of relapse (95). Indeed, smokers at risk for relapse showed more insula activation while processing smoking-related words (95). In individuals with dependence to cocaine, hyperactivity to drug-specific stimuli contrasts with abnormally low activity when self-regulation processes are engaged [e.g., inhibition; (96)].

Other evidence supporting the important role of the insula in addiction processes arise from human brain lesion studies. Strokes that damage the insular cortex tend to literally wipe out the urge to smoke in some individuals previously addicted to cigarette smoking (12). In this study, smokers with brain damage involving the insula were more likely to quit smoking easily and immediately, without relapse and without a persistent urge to smoke (12). These results support a novel conceptualization of one of the mechanisms by which the insula participates in maintaining addiction.

The insular cortex (and most likely the anterior insula) responds to interoceptive signals (due to homeostatic imbalance, deprivation state, stress, sleep deprivation, etc.). Besides the translation of these interoceptive signals into what may become subjectively experienced as a feeling of “urge” or “craving,” we hypothesize that the insular cortex activity increases the drive and motivation to smoke (or take drugs or to gamble) (a) by sensitizing or exacerbating the activity of the habit/impulsive system; and (b) by subverting attention, reasoning, planning, and decision-making processes to formulate plans for action to seek and procure cigarettes or drugs (97). Put differently, these interoceptive representations have the capacity to “hijack” the cognitive resources necessary for exerting inhibitory control to resist the temptation to smoke or use drugs by disabling (or “hijacking”) activity of the prefrontal (control/reflective) system.

Stress could be a critical candidate for understanding the impact of abnormal level of proprioception on impulsive and reflective systems (98, 99). Indeed, whereas at low to moderate levels of stress, the reflective system’s activity may be enhanced, abnormally high levels of stress tend to attenuate activity within the reflective system (99). This may be applied to alcohol use by successfully inducing drug craving in the laboratory through the generation of stress (e.g., 5-min individualized guided imagery exposure of each participants’ own recent stressful scenarios) (100). Research has emphasized the existing association between exposure to stress, abnormal persistent level of changes in the body state (heart rate, salivary cortisol levels), persistent craving, and use of compulsive individuals’ preferred drug [for a review, see Ref. (101)]. Interestingly, alcoholics’ prefrontal and striatal-limbic regions (including the insula) are hyper-responsive to neutral-relaxing condition, making activation pattern between neutral relaxed and stressful cues indistinguishable (102). This result suggests a dysregulated response to stress that induces craving and increases the risk of relapse in alcoholics (101).

### The impact of poor metacognition on addictive behaviors

Recent theoretical accounts (17, 103) advance that a dysfunction of the interoceptive system may also hamper self-awareness, which could take the form of failure to recognize an illness (i.e., lack of insight). Indeed, perceived need for treatment concerns only a minority of individuals suffering from addiction (104), which might reflect dysfunction in cognitive processes and the neural circuits underlying self-awareness (103). The underestimation of the addiction severity might drive these individuals’ excessive drug use, where control of use becomes exceedingly deregulated.

Impaired insight ability could be estimated through the evaluation of metacognition capacity, which refers to our ability to discriminate correct from incorrect performance. The presence of impaired metacognitive capacities in substance addiction has received support from previously identified dissociations between subjective and objective markers of behavior in addicted individuals (54, 105–111). For example, Brevers et al. (105) showed that pathological gamblers were impaired in their capacity to evaluate accurately the quality of their decisions during an artificial grammar-learning paradigm, in which the quality of choice remains uncertain throughout the task. Specifically, after each trial of this task, participants had to indicate how confident they were in their grammaticality judgments. Results showed that, by contrast with their controls, there was no correlation between pathological gamblers’ grammaticality judgments and their level of confidence, which suggests a disconnection between performance and confidence in pathological gamblers. Additional evidences for lowered metacognitive capacities in addicts comes from a study by Hester et al. (109) who observed diminished commission error awareness during a go/no-go response inhibition task in chronic cannabis users. In addition, these authors observed that the insensitivity of chronic cannabis users to detect errors was associated with the absence of right insula activity during unaware errors (when compared with aware error activity). Furthermore, lower levels of insula activity were correlated with higher levels of recent cannabis use, and higher levels of cannabis craving were associated with poor error-awareness rates. These results are in line with hypotheses suggesting that insula dysfunction may contribute to impaired interoceptive awareness and heightened experience of drug-related urges, potentially at the expense of other interoceptive signals necessary for advantageous decision-making and the ability to learn from errors (13, 112).

Other studies have also observed dissociations between subjective and objective markers of performance when they were separated in time, i.e., when subjects were asked to evaluate their overall performance after completing the experimental task in its entirety (54, 108, 111). For instance, Goldstein et al. (54, 108) observed that healthy control participants showed incentive-related performance enhancements during a sustained attention task, and that these improvements in performance correlated with their self-reports of task engagement. These same correlations did not reach significance in the cocaine subjects. Moreover, these investigators observed that healthy control subjects, but not cocaine subjects, showed significant correlations between monetary-driven P300 amplitudes and their respective behavioral performance responses (108). More recently, Moeller et al. (111) showed that, in cocaine addicted individuals, there was a poor correspondence between the most selected picture category on a probabilistic choice task (that assessed objective preference for viewing four pleasant, unpleasant, neutral, and cocaine pictures) and self-reported most chosen picture category (ascertained at the conclusion of the probabilistic task).

Finally, additional studies have also focused on the impact of diminished metacognitive capacities on addicts’ forthcoming decisions or performances (106, 107, 110). For example, Le Berre et al. (110) observed dissociations between the predictions of future recognition performance (estimated with self-rated ability to perform adequately a following word-recognition task) and actual recognition performance in alcohol-dependent individuals. More recently, Brevers et al. (106) examined metacognitive capacities in a sample of pathological gamblers by asking participants to wager on their own decisions after each choice during their performance of the Iowa Gambling Task [i.e., IGT with post-decision wagering; Ref. (113)]. These authors observed that, unlike healthy controls, pathological gamblers tend to wager high while performing poorly on the IGT (i.e., choosing option featuring high rewards but higher losses rather that options featuring low rewards and losses). This result suggests that pathological gamblers exhibited impairments not only in their ability to correctly assess risk in situations that involve ambiguity, but also in their ability to correctly express metacognitive judgments about their own performance. That is, pathological gamblers not only perform poorly, but they also erroneously estimate that their performance is much better than it actually is. In chronic cigarettes smokers, Chiu et al. (107) examined whether or not fictive prediction errors (i.e., when fictive outcomes contradict with the expectation) during a non-active gambling choice task impacted smokers’ forthcoming choice. These authors observed that the fictive prediction error was computed by chronic smokers (indexed by a corresponding signal change in the ventral striatum) but did not influence their behavioral choices (when compared with control participants). Findings from these studies (106, 107, 110) further support hypotheses advancing that a dissociation between objective and subjective markers may contribute to impaired decision-making and the ability of failure to learn from errors that underlies perseverative behavior.

This abnormal degree of dissociation found in addicted people between the “object” level and the “meta” level, raised the possibility that poor metacognition leads to poor action and decision-making monitoring and adjustment (114). However, much remains to be done in order to identify how different regions of the prefrontal cortex interact with interoceptive signals to promote accurate judgment performance and further cognitive control on decision-making, memory as well as on the sense of agency in healthy participants (115) and in addicts (17). Anatomically, the insula is a primary site for receiving interoceptive signals, but in turn the insula is connected to widespread regions of the prefrontal cortex, and hence this interoceptive-prefrontal interaction may be mediated by the insula (17, 116).

## Lack of Willpower in Addicts; the Chicken or the Egg Question

### Effects of acute drug administration on willpower capacities

Evidence from cognitive and behavioral sciences has led to the idea that acute drug primes limbic incentive neurocircuitry (striatum, amygdala, insula, mesencephalon) while suppressing top-down supervisory control circuits [for a review of alcohol studies, see Ref. (117)]. Of particular interest is the altered capacity to detect and resolve errors made on a variety of cognitive tasks [for a review, see Ref. (62)] and also, when subjects are given alcohol, they tend to take more risks which parallels increased activation in the striatum to risky compared with safe choices and the neural response to notification of both wins and loses are dampened throughout NAcc, caudate, thalamus, and insula (118).

In addition, emotional processing could be dramatically altered after the ingestion of some drinks as illustrated by some recent studies showing a lack of amygdala (119) and of insula (120) recruitment by threat-indicating faces during alcohol intoxication; thus making threatening and uncertain outcomes in social context less of a moderator in inebriated participants.

Together, research on the acute effects of drugs on brain and cognitive processes is compatible with the idea that it compromises one or more of these brain systems discussed in the present paper, a disruption that may prevent the efficient exercise of willpower. However, in most of the studies, these brain abnormalities are not associated with abnormal behavioral pattern (e.g., low working memory performance) [but see Ref. (121) for abnormal both pattern of activation and behavior consecutively of alcohol ingestion], thus raising the question of the significance of these hypo-/hyper-activation found in neuroimaging studies [see (117) for a discussion of this issue]. Although not demonstrated yet, one could expect that these brain changes would be more sensitive measures than behavioral ones (122), which is likely to become abnormal on more demanding tasks.

### Vulnerabilities in the decision process and the risk of addiction

Vulnerability to substance use and gambling disorders encompasses multiple dimensions of the individual’s life, ranging from biological factors to broader environmental and cultural variables [e.g., Ref. (123)]. Particularly sensitive is the period of adolescence starting with the biological changes of puberty and ending with the time at which the individual attains a stable, independent role in society. During this period decision-making is increasingly independent of adult influences as well as more vulnerable because of an enhanced taste for risk together with impulsive actions and decisions that can sometimes lead to serious consequences. This is a context that makes peers influence more powerful (for better or for worse) and novel experiences more attractive. Thus one would not be surprised to observe that adolescence is an experimentation period with a number of exciting experiences, e.g., alcohol, drugs, and gambling. Note that initiation is not a pathological process *per se* but sometimes it may evolve into severe disorders (i.e., compulsive behaviors) (124). At the extreme, this state of “inflexibility” exemplified by drug and non-drug addictive behaviors has been thought to reflect impaired “basic” behavioral learning processes, poor self-regulation, and impaired decision-making [e.g., Ref. (4)]. As a consequence, “willpower” may be too limited. Thus, one characteristic of adolescence is that the power of positive short-term consequences of choice and behaviors may exceed self-regulation capacities, which can be risky to health, yet might be normal within adolescent social development [Ref. (125); for a review, see (126)]. Individual motivational and regulatory responses are thought to increase the risk of alcohol and gambling misuse in adolescents. However, individual differences presented far before the onset of puberty are also important to understand the elevation of risk [e.g., low capacities of delay of gratification in children at age 4 predict cocaine/crack use in individuals vulnerable to psychosocial maladjustment, (127)]. As another example, higher levels of impulsive behavior as early as age 3 predict aggressive behavior and drug use in adolescence (128). Thus, the quality of pre-adolescents’ cognitive and affective functioning (e.g., resisting immediate temptations) is important to take into account in order to investigate the addictive risk in adolescents (129). However, during adolescence, demanding context (e.g., peer influence) and sensation seeking are rising dramatically; thus encouraging experimentation with novel behaviors, which also require “willpower” to prevent a number of deleterious consequences.

It remains difficult to know whether these abnormalities are a direct result of the toxic effects of alcohol on the brain (i.e., the consequence of use) or whether pre-existing brain conditions (antecedent to the initiation of use) are present that differentiate those who begin using at a young age form those who do not (130). The “chicken or the egg” question has been addressed in different ways. For instance, in adolescent girls, more drinking days in the past year predicted a greater reduction in performance on visuospatial memory tasks, whereas in boys, more hangover symptoms in the year before follow-up testing predicted worsening of sustained attention (131). These results suggest that due to neurotoxic effects, alcohol-use disorders during adolescence (including associated withdrawal symptoms) may interfere with normal brain maturation. However, despite increased frequency of use over a 4-year follow-up period in a community-based sample or early adolescents (132), working memory among participant remained stable over the 4-year period suggesting that the detrimental influence of increased drinking frequency on working memory is marginal between early and mid-adolescence.

Another way to address this issue was to compare adolescents who have no or minimal alcohol/drug exposure, but who have a positive family history (FH) for alcoholism (FH+), to age-matched adolescents who have a negative FH− for alcoholism. The FH+ group is an ideal genetic-risk model, as a positive history of alcoholism is associated with an earlier onset and higher magnitude of use as well as with a higher prevalence of alcohol-use disorders in adolescents and young adults. The neuropsychological literature found mixed results regarding cognitive impairments in FH+ prior to the initiation of alcohol consumption. Evidence shows that FH+ youth have deficits in abstract reasoning and planning, and they have lower IQ scores, poorer academic performance, and slower trajectories in cognitive improvement at 1-year follow-up when compared to FH− youth (133). However, other studies failed to document differences between FH+ and FH− youth. Interestingly, deficits in FH+ youth were predominantly found in children of antisocial alcoholics (134), which may constitute a critical and poorly controlled factor to explain discrepancy across studies. In addition, the number of relatives determining the criteria for a positive FH of substance (a single parent in some studies, numerous in others) is also likely to explain differences across studies. Indeed, a greater family loading of alcoholism may be associated with a greater genetic susceptibility for developing a substance-use disorder than a lesser degree of family loading.

From a neurobiological perspective, FH+ youth exhibit smaller overall total brain volumes than their FH− counterparts and less inhibitory frontal activation during the performance of a go/no-go functional magnetic resonance imaging task, compared to FH− comparison subjects (135). At the structural brain level, information processing speed was correlated significantly with white matter volume in FH− females only (136), which may indicate that subtle abnormal cognitive/brain relationship could represent a risk factor for substance abuse in female adolescents who have not yet initiated drug use.

A great number of researches support the idea that higher impulsivity, which could be defined as “actions which are poorly conceived, prematurely expressed, unduly risky, or inappropriate to the situation and that often result in undesirable consequences” (137), elevates the risk of addiction in young population. For instance, slower rates of development of behavioral self-control strongly and specifically predict early initiation of drug use at 14-year-old and higher number of drug-related problems at 17-years old (138). In line with this result, impulsive/hyperactive symptoms of ADHD in children were the stronger predictors in a prospective study for initiation of tobacco, alcohol, and illicit drug use at 14 (139). In another group with genetic risk of addiction, the adolescent offspring of substance-use disorder parents showed elevated prevalence of alcohol-dependency and stimulant use (140). This relationship is hypothesized to be due to impulsivity-related endophenotype (141). In the same vein, impulsivity measures (personality dimension of constraint) during adolescence also significantly predict problem gambling behavior at a follow-up assessment [e.g., Ref. (142)].

In sum, during childhood and adolescence, decrements in cognitive abilities, associated with familial history of substance abuse (and antisocial personality component and multiplex FH of alcoholism), may be present prior to cognitive impairments that result from early initiation and continued use of alcohol and drugs.

## From Neurocognitive Theory to Clinical Practice

Based upon the model proposed in this article, a number of clinical and cognitive interventions are thought to improve treatment outcomes of addictive behaviors. These interventions generally aim at targeting one subsystem at a time from the triadic neural system model of willpower presented in this article. One strategy may be fooling the impulsive system by desensitizing automatic attention toward addiction cues, positive implicit memory associations with addiction cues, and approach tendencies toward addictive object. Boosting self-regulation control along with boosting metacognition and self-awareness of the cognitive limitation, and reducing interoceptive signaling of homeostatic disturbances are also key strategies to alter addiction-related choices and actions. Applications of some of these strategies are already taking place, such as boosting the capacity of the reflective system through physical exercise and mindfulness, through which researchers aim to modulate how an individual processes and integrates afferent sensing from the inside of the body (14). Although interesting, we argue that clinical interventions rooted in the qualitative description of abnormal interactions between the three above mentioned systems could lead to more robust and significant changes. Importantly, our defended clinical approach considers the necessity to adapt clinical strategies to every addicted individual. Indeed, there is evidence showing that these participants are highly heterogeneous with respect to cognitive and brain determinants of their addictive behaviors [for pathological gamblers, see Ref. (143); for polysubstance abusers, see Ref. (144)]. For instance, the principle of equifinality (a give end state – e.g., compulsive drug use – can be reached by many potential pathways) is particularly clear in case of impulsivity, a concept that encompasses a number of aspects including impulsive, reflective and proprioceptive determinants (14, 145).

### Strategies aimed at “fooling” the impulsive system

Based on an early remarkable proposition made by MacLeod et al. (146), one of the most widely used cognitive bias modification targets attention approach. In this article, the detection of the probe appearing distally from the negative information results in attenuating the intensity of anxiety and depression reactions to subsequent laboratory stressor as well as to stressful life events [see, Ref. (147)] in healthy participants. When applied to alcohol abuse disorders, it has recently been demonstrated that, when randomly assigned to either the attending (i.e., directing attention toward the location of alcohol-related cues) or avoiding (i.e., directing attention away from the location of alcohol-related cues) condition of a modified probe task, only alcohol-dependent participants who were required to systematically avoid alcohol cues showed swifter therapeutic progress with discharge from the program gaining 28 days on average and reduced risk of relapsing (time to relapse was significantly 1.25 months longer) following the attentional bias modification training intervention (148).

Similar findings were obtained with attempts to change approach tendencies toward alcohol cues (149, 150). Specifically, studies have shown that training abstinent individuals with alcoholism to make avoidance movements in response to alcohol pictures induced better treatment outcomes (decreased alcohol relapse rate at 1 year follow-up) (149, 150). Importantly, older patients and patients with a strong approach-bias profited most from this training sessions (149). Of note, in individuals with alcoholism, alcohol relapse rates increase as the relative tendency to avoid alcohol increases, thus suggesting that an avoidance orientation toward alcohol can potentially be harmful in clinical samples (51).

With regard to implicit memory bias toward addiction-related cues (e.g., good/bad; sedative/arousal), Houben et al. (151) showed that, following an evaluative conditioning session (in which alcohol-related cues were consistently paired with negative stimuli), problem drinkers showed stronger negative implicit attitudes toward alcohol and diminished their alcohol consumption within a week after the evaluative conditioning session.

### Strategies aimed at boosting the “reflective” system

Experimental procedures aimed at improving self-regulatory processes could also have been used to modify substance-taking behaviors. For instance, by experimentally priming either disinhibited (i.e., participants were told that they should try to inhibit responding to “Stop” stimuli if possible, but that this was less important than rapid responding to the “Go” stimuli) or restrained behaviors (i.e., participants were told that they should try to respond quickly to “Go” stimuli if possible, but that this was less important than successful inhibition on “Stop” trials) while participants completed a Stop-Signal task, Jones et al. (152) found that increased consumption of beer during a test phase in the “disinhibition group,” as compared to the “inhibition group.” Similar results have been found when comparing alcohol use during a taste phase after experimentally building an association between a “go” response and alcohol cues (by using an alcohol version of the go/go-go task) compared with a condition in which the “no-go” response was associated with alcohol cues (153).

As another recent important finding, Verbruggen et al. (154) demonstrated that proactive inhibitory control may have a direct impact on risk-taking. In this study, participants were presented with six free-choice options on every trial. Each option was associated with a certain amount to win; however, participants were informed that the higher the amount, the less probable a win. In some blocks (i.e., stop condition), in addition to gambling choice, participants were required to stop the planned manual choice response when an occasional signal occurred. Results indicated that participants reduced risky gambling in the stop condition when compared to non-stop condition. Hypothetically, the stop condition induced a proactive motor responding, that is, a general state of cautiousness that may have enhanced cognitive control and in turn reduced risky decision-making. In other words, when individuals are preparing to stop, people make proactive adjustments and become more cautious in executing motor responses (155–158), which in turn may diminish the influence of automatic, emotionally driven processes associated with high and uncertain rewards. Altogether, these studies advance the evidence for a “control transfer” between cognitive domains, which could open new avenues for intervention aiming at reducing addictive behaviors in a clinical population. Unfortunately, a recent study found that the gain on cautiousness up to 2 h when making decisions becomes negligible 24 h later (159). This finding underlines the need to find better parameters of inhibition training to achieve clinical efficacy.

### Strategies aimed at controlling “urges” and interoceptive signaling

In this paper, we emphasized that the increased liability for substance use may also emerge from a highly embodied experience through the insular cortex. This process may overwhelm the cognitive control system by providing a highly emotionalized experience (e.g., intense state of substance-related craving) and by sensitizing substance abusers to the conditioning of interoceptive drug effects (5, 93). Therefore, these individuals might benefit from interoception-modifying techniques, such as mindfulness exercises, biofeedback, or interoceptive exposure therapy, in order to train reappraisal of the significance of bodily feedback triggered by addiction-related cues (93). In addition, because dysfunction of the interoceptive system may also hamper self-awareness (17, 103), individuals suffering from addiction might also benefit from intervention aiming at enhancing insight. For instance, in recently abstinent alcohol-dependent patients, Jung et al. (160) showed that a brief insight-related intervention (five sessions in 2 weeks; 15 min per session aiming at promoting conscious awareness of symptoms associated with alcohol use) enhanced participants’ level of motivation to change their alcohol-related behavior. In another study, Kim et al. (161) showed that the rate of (1-year) abstinence among alcohol-dependent patients appears to be significantly heightened by their post-cure level of insight. These results suggest that a high level of insight has a positive effect on the diagnostic process, motivation for treatment, and substance-related behavioral change, and may play a critical role in the recovery process.

### Triadic model-based strategies

As mentioned above, the equifinality concept of addictive behaviors suggests the need to investigate a personalized clinical approach instead of the standard diagnostic-based view. Concretely, it is plausible that based on a cognitive assessment investigating the strength of approach tendencies triggered by either negative emotions (negative reinforcement), positive emotions (positive reinforcement) or both and given the individual’s efficiency of supervisor functions and the contribution of a particular state of feeling leading to drug use or behavioral addictions, one may benefit more from boosting response inhibition control under stress-induced and craving conditions than another who will gain more control over his/her compulsive behaviors by undergoing positive emotion induction while exercising inhibitory control. For these subjects with high levels of reported craving for drugs or gambling, it sounds reasonable to include mindfulness sessions, which obviously alter the subjective experience, possibly by disconnecting craving-related regions such as the insula and the ventral striatum [for smoking craving, see Ref. (162)].

It is obvious that the assumed necessity to work on the association of systems rather than on one single system needs a lot of development and empirical supports. At this point, our suggestion is supported by (1) a vast research literature showing that maladaptive processes to drug and non-drug behaviors are numerous and effect bottom-up/automatic as well and top-down/intentional registers; (2) the high heterogeneity of clinical profiles (positive reinforcement, negative reinforcement, habits, impulsiveness, craving intensity, automatic cue-induced reactivity, and so on) and of cognitive determinants of these learning processes; (3) the idea that supervisory function fluctuate over time and context (e.g., drug or gambling use, stress, cue exposure) [see for a discussion, Ref. (163)].

Cognitive based interventions have already proven their efficacy in reducing craving and alcohol use, delaying relapse, and improving therapeutic commitment. It is not clear yet which particular combination between pharmacotherapy (e.g., naltrexone, acamprosate), brain stimulation, and cognitive training best fit the different forms of addiction profiles (e.g., sensation seekers/positive reinforcement vs. harm avoidant/negative reinforcement).

Based on the proposed triadic model, predictions of an improved treatment strategy would be to deliver the magnetic brain stimulation in such a way to (1) downregulate (or block) the insula; (2) upregulate (or stimulate) the prefrontal cortex. Upregulation of the dorsolateral prefrontal cortex by high frequency transmagnetic brain stimulation has already demonstrated its positive outcomes on nicotine dependence [for a review, see Ref. (164)]. For instance, this strategy reduces the power of smoking-related cues to elicit craving. However, a number of other brain regions could be indirectly altered and further studies are needed to better characterizing those motivational-related brain regions altered by upregulation of the dorsolateral prefrontal cortex.

## Conclusion

The discovery of the insula as a important brain structure specifically in smoking addiction does not undermine the seminal work generated to date on the roles of other components of the neural circuitry implicated in addiction, and impulse control disorders in general, especially the mesolimbic dopamine system (incentive habit system), and the prefrontal cortex (executive control system). Addressing the role of the insula only complements this prior work, leading researchers to investigate how inter-connected activities between the prefrontal cortex, the striatum-amygdala system and the striatum accounts for distinct dimensions of clinical phenomenology of addictive behaviors. At the cognitive and behavioral level, various aspects of sensitized automatic stimulus-driven processing associated with poor self-control capacities elevate the likelihood that a state of addiction be perpetuated, particularly in the context of stress and of addiction-cue exposure.

With respect to clinical interventions, our recommendation is to adapt treatments to each individual’s triadic system configuration by adopting a multidimensional approach, focusing on the interactions between automatic, intentional, and proprioceptive cognitive processes involved in the risk of developing an addictive behavior (in vulnerable populations), in the maintenance of such behaviors (in addicted people) or in the risk of relapse (in patients). While neuropsychological testing has advanced tremendously with respect to brain areas such as the prefrontal cortex (or measuring executive functions), there is still a great lag in neuropsychological evaluation of those state-dependent systems (such as the insula). Measuring strengths or weaknesses in these systems could be the next great advancement in neuropsychological evaluation.

This effort will undoubtedly allow the discovery of novel therapeutic approaches for treating several impulse control disorders, including breaking the cycle of addiction.

## Conflict of Interest Statement

The authors declare that the research was conducted in the absence of any commercial or financial relationships that could be construed as a potential conflict of interest.
